# Secondary Analysis of a Dataset to Estimate the Prevalence of Vertebral Subluxation and Its Implications for Health Promotion and Prevention

**DOI:** 10.7759/cureus.48755

**Published:** 2023-11-13

**Authors:** Christie Kwon, Jose N Binongo, Matthew McCoy

**Affiliations:** 1 Department of Chiropractic Sciences, Life University, Marietta, USA; 2 Board of Directors, Foundation for Vertebral Subluxation, Kennesaw, USA; 3 Department of Biostatistics, Emory University Rollins School of Public Health, Atlanta, USA

**Keywords:** prevention science, prevalence, epidemiology, chiropractic, vertebral subluxation

## Abstract

Background

Vertebral subluxation (VS) is a clinical entity defined as a misalignment of the spine affecting biomechanical and neurological function. The identification and correction of VS is the primary focus of the chiropractic profession. The purpose of this study is to estimate VS prevalence using a sample of individuals presenting for chiropractic care and explore the preventative public health implications of VS through the promotion of overall health and function.

Methodology

A brief review of the literature was conducted to support an operational definition for VS that incorporated neurologic and kinesiologic exam components. A retrospective, quantitative analysis of a multi-clinic dataset was then performed using this operational definition. Descriptive statistics on patient demographic data included age, gender, and past health history characteristics. In addition to calculating estimates of the overall prevalence of VS, age- and gender-stratified estimates in the different clinics were calculated to allow for potential variations.

Results

A total of 1,851 patient records from seven chiropractic clinics in four states were obtained. The mean age of patients was 43.48 (SD = 16.8, range = 18-91 years). There were more females (n = 927, 64.6%) than males who presented for chiropractic care. Patients reported various reasons for seeking chiropractic care, including, spinal or extremity pain, numbness, or tingling; headaches; ear, nose, and throat-related issues; or visceral issues. Mental health concerns, neurocognitive issues, and concerns about general health were also noted as reasons for care. The overall prevalence of VS was 78.55% (95% CI = 76.68-80.42). Female and male prevalence of VS was 77.17% and 80.15%, respectively; notably, all per-clinic, age, or gender-stratified prevalences were ≥50%.

Conclusions

To date, this is the first study of its magnitude and application of an operational definition to estimate the prevalence of VS. Albeit nonrandom, the sample had a broad geographic distribution. The results of this study suggest a high rate of prevalence of VS in a sample of individuals who sought chiropractic care. Concerns about general health and wellness were represented in the sample and suggest chiropractic may serve a primary prevention function in the absence of disease or injury. Further investigation into the epidemiology of VS and its role in health promotion and prevention is recommended.

## Introduction

Background and theoretical framework

Established in 1895, the chiropractic profession continues to explore the establishment of an operational definition of vertebral subluxation (VS) that is evidence-based. VS is defined by chiropractors as misalignment of the spine affecting biomechanical and neurological function; both body systems must be involved [1]. The spinal column houses and protects the spinal cord and nerve roots. According to Gray’s Anatomy, “the nervous system controls and coordinates all organs and structures of the human body” [2].

Although the assessment and correction of VS are the basis of chiropractic, admittedly there is a lack of gold standard within the profession as to what constitutes VS [3]. The term subluxation and its neurological consequences have been explored in the chiropractic and medical literature for more than a century [1]. The VS model was strengthened in the 1980s and 1990s as the neurological and physiological effects of biomechanical misalignment were further studied [4]. In this same period of time, multiple models of VS have been developed, and controversies over the operational definition of VS have since ensued. Despite a lack of agreement on the best direct measurements to evaluate for subluxation, research has demonstrated that a number of methods of detection are reliable and valid and could be incorporated into an operational definition for VS [3].

An operational definition clarifies the criteria for assessing a problem. The operational definition is a concrete description of a construct in terms of the variables, procedures, actions, or processes by which it could be measured or observed [5]. Adoption of an operational definition of VS is necessary to support scientific knowledge and a coherent understanding of the criteria to determine whether a person is subluxated. Moreover, the selection of an appropriate operational definition affects the validity of subsequent prevalence calculations. Biomechanical assessment is commonly conducted by measurement on plain film radiographs, but neurological components are measured by a variety of valid tests. Various reliable exam findings suggest neurological disruption, such as inflammation of the C2 (second cervical vertebra) dorsal root ganglion (DRG), positive Fakuda Step test, leg length inequality, the tautness of the erector spinae muscles, or upper extremity muscle weakness.

The presence of any of these findings, coupled with misalignment of vertebrae as determined by X-ray analysis, are directly indicative of VS and will be considered as the operational definition for the purpose of this study. This supposition is consistent with the practices of the majority of chiropractors [3], with palpation and leg length inequality being the most commonly reported VS indicators in the published literature, and consideration of multiple criteria for the neurological components of VS reduces the likelihood of false-positive or false-negative findings. As stated by Kent, practitioner flexibility is aided by the vast criteria available to identify and correct VS [1]; and research components that align with clinical practice procedures facilitate the implementation of evidence-based practice [3].

Problem statement

Chiropractors maintain that the presence of VS can lead to negative health outcomes [6]. The Association of Chiropractic Colleges defines subluxation as “a complex of functional and/or structural and/or pathological articular changes that compromise neural integrity and may influence organ system function and general health” [7]. Yet, to date, there have been no large epidemiological studies that attempted to quantify the prevalence of VS within the general population.

There is, therefore, a need to estimate the prevalence of VS in the general population based on the presence of biomechanical and neurological indicators. A high prevalence of subluxation may suggest a potential public health issue and preventative measures should be aimed at the reduction of these indicators. Having this information would be beneficial to the general public as well as the chiropractic profession.

Purpose statement

The purpose of this study is to estimate the prevalence of VS based on neurological and radiographic indicators in patients presenting for chiropractic care from a multi-clinic dataset. Age and gender-stratified prevalence in each clinic will also be calculated. This analysis will provide a basis for primary prevention measures relevant to VS and its implications for health promotion and prevention.

Review of literature

Clinical Significance of VS

The primary premise of the chiropractic profession is that the body is a self-maintaining, self-healing organism and that reducing and/or correcting VS allows the nervous system to function better and allows the fullest expression of life [6]. The case history and examination findings together provide evidence for VS at specific spinal levels.

Any number of tissues, organs, or body systems can be impacted by misalignment at various levels of the spinal column based on the vast distribution of the peripheral nervous system, as well as its relationship to other body systems. The most commonly reported reasons for people attending chiropractic care have been noted as (median) low back pain (49.7%; interquartile range (IQR) = 43.0%-60.2%), neck pain (22.5%; IQR = 16.3%-24.5%), and extremity problems (10.0%; IQR = 4.3%-22.0%) [8]. It is important to note that the presence of VS does not require any associated symptomatology to be present; therefore, the evaluation of asymptomatic individuals may result in positive findings as well. Outcomes related to musculoskeletal or non-musculoskeletal complaints are important but not necessarily indicators of correction to a specific VS. A review by Russell notes an increased demand for chiropractic care by patients of all ages for “wellness/prevention,” general health, and maintenance of spinal health [3]. The benefits of VS correction are well beyond analgesic effects and constitute a broad spectrum of salutogenic and health-promoting outcomes [1]. The literature supports the role of chiropractic in affecting quality of life and overall health. Researchers conducted a survey study of 2,818 adult patients in 156 chiropractic clinics from the United States, Canada, Australia, and Puerto Rico [9]. They found that in addition to physiological changes recorded by the chiropractors, patients self-reported significant improvements in four domains of health (physical state, mental/emotional state, stress evaluation, life enjoyment) and quality of life over one to three months to three years with chiropractic care.

The negative health consequences of VS warrant further exploration [6], but it is important to first estimate the prevalence of VS. Smaller studies have attempted to answer this question and reported subluxation prevalence ranging from 90% to 99.4% in the chiropractic literature [10,11] and 35-95.1% in orthopedic papers [12,13]; conservative management methods are recommended in most of these cases. Medical subluxation was diagnosed based on radiographic measurements alone [12,13]. One study’s operational definition considered neurologic (leg length inequality and palpation) findings without mention of radiographs [10]. Current prevalence estimates of VS are anecdotal. In this study, a more comprehensive operational definition is attempted to estimate the prevalence of VS in a large sample from a multi-site clinical setting.

Methods of Evaluation for VS

The purpose of the chiropractic examination is to assess the presence of VS through the measurement of its components. There is no one variable to measure VS; rather, a combination of several variables is used to identify subluxation. Furthermore, there is not a consistent combination of variables agreed upon by all chiropractors; however, there is general agreement on the need to assess neurologic and kinesiologic components. Several direct and indirect indicators of VS are identified in the literature [3]. Direct indicators, such as those employed in this analysis, provide information on where to apply a chiropractic adjustment and follow-up information on whether the VS was corrected. Neurological dysfunction has been traditionally detected through a number of means including static and motion palpation, such as for inflammation and taut-/tenderness, evaluation for leg length inequality, or manual muscle testing (MMT). More specific tests, including the Fakuda Step test, are used to evaluate the integrity of the cerebellum or dorsal column tracts of the central nervous system. Radiographic evaluation is also used to ascertain the biomechanical component of the VS; imaging allows the practitioner to measure this misalignment from multiple angles. “Common to all concepts of subluxation are some form of kinesiolog[ic] dysfunction and some form of neurologic involvement,” according to Lantz [14]. Examination findings elucidate the presence and location of VS. However, it is also recognized that VS may have physiological manifestations that are not yet symptomatic.

Specific examination components of the operational definition used in this study included (1) inflammation of the C2 (second cervical vertebra) DRG, (2) leg length inequality, (3) tautness of the erector spinae muscles, (4) upper extremity muscle weakness, (5) Fakuda Step test, and radiographic analysis based on the (6) frontal atlas cranium line and (7) horizontal atlas cranium line. Each of these direct measurements for VS has been discussed in the published literature heretofore and has shown moderate to high reliability and construct validity [3].

Methods of Analysis for Neurological Dysfunction

Static and motion palpation are used to assess for tenderness, stability, and motion between spinal segments. It assesses the bony anatomy and surrounding soft tissues. Taut and tender fibers, hypermobile segments, hypomobility, or myospasm could all be suggestive of VS. Bony and soft anatomy of the cervical and lumbar spines can be evaluated in this manner. Moderate to high reliability and construct validity of palpatory findings, including tenderness, are noted in the literature [3].

The cervical spine is palpated to evaluate motion segments and note any tenderness. An inflamed C2 DRG, located between the first (C1) and second (C2) cervical vertebrae, is a common sign of nerve entrapment. Due to the small space between C1 and C2, cadaver studies have revealed that this nerve root is highly susceptible to entrapment [15]. Inflammation in this region of the cervical spine can lead to headaches or areas of paresthesia.

Neurological disruption at a spinal level can cause reflex misfiring that results in postural distortions such as functional scoliosis or imbalance of the legs. Leg length inequality is a key component of the chiropractic examination and one of the most commonly used signs of spinal imbalance. Inter and intraexaminer reliability of leg length assessments has been established by extensive studies among chiropractors [16,17].

The erector spinae muscle group is a set of three muscles that run vertically from the base of the skull down the length of the spine and aid in the extension of the torso. Tautness and asymmetry of these muscles have been associated with the presence of a functional short leg [18], and these two findings collectively support the presence of subluxation.

MMT has shown good reliability and validity for the evaluation of neuromusculoskeletal dysfunction [19]. Research suggests that MMT can reveal central or peripheral nervous system issues assessed by both chiropractors and physical therapists. “Manual muscle tests evaluate the ability of the nervous system to adapt the muscle to meet the changing pressure of the examiner’s test,” according to Cuthbert and Goodheart [19]. Muscle weakness suggests inhibition of anterior horn motor neurons as associated with spinal dysfunction. Upper extremity muscle weakness coupled with head rotation is used as an examination procedure in this study.

The Fakuda (aka Unterberger’s) Step test is a special neurological test used to evaluate for vestibular system-related balance problems. It is commonly associated with vertigo or dizziness. The test measures rotation to the side of the lesion while the patient marches in place with eyes closed. The reliability of this exam procedure in determining the side of the lesion has been questioned, particularly in acute cases [20], but this procedure is still widely used in clinical practice.

Methods of Radiographic Analysis of Biomechanical Dysfunction

Plain-film radiographic imaging is thought to be highly reliable, and imaging is the most objective way to assess the biomechanical component of VS. There is a high level of agreement for the use of diagnostic imaging in chiropractic practice [21]. The algorithm of this study focuses on imaging the cervical spine to determine misalignment based on three-dimensional analysis [22]. Orthogonality of 90 degrees is considered to be the ideal alignment for the cranium in relation to the cervical spine. The frontal atlas cranium line (FAC) measurement determines the laterality (right or left-sided) component of the first (C1) cervical vertebra misalignment along the X-axis plane of the body. The horizontal atlas cranium line (HAC) is a measure of the anterior or posterior misalignment component along the Z-plane with reference to the FAC. Genetic abnormalities and asymmetries can influence these measurements, so cutoff values of >0.25 degrees are commonly considered indicative of subluxation. Measurements are considered to be accurate to 1/100th of a degree [23].

## Materials and methods

We utilized a dataset of 3,364 patient records from 12 chiropractic clinics located in 11 different U.S. cities for this retrospective analysis. Data were collected during initial patient exams from March 2009 to October 2019 and recorded in the proprietary SONUS central data repository. This study attempted to accomplish the following three aims: (1) develop an operational definition of VS, (2) apply the operational definition to estimate the prevalence of VS in patients presenting to a network of chiropractic clinics, and (3) evaluate differences in prevalence by age and gender.

Variables of interest included demographic information (date of birth, sex), chief presenting complaints (primary reasons for seeking care, current and chronic symptoms, motor vehicle accident history), spinal radiograph data for biomechanical assessment, and evaluation of five indicators of neurological function.

Analysis was limited to clinics that recorded initial exam findings and adult patients (≥18 years of age). Subjects for whom age could not be calculated due to data entry errors were excluded (n = 132). Minors (n = 221) were not included in the data analysis because it is possible that prevalence is strongly impacted by age, and children’s physiology is quite different from that of adults.

SONUS is the central data repository system utilized by this network of clinics [23]. This proprietary database is used to record de-identified patient history information, examination findings, and follow-up data over the course of chiropractic care. Each variable in SONUS is recorded as “Right,” “Left,” or “Both” if positive; negative data points were left blank. Initially, the data from SONUS had to be imported into SAS. Variables were converted to dichotomous values, 1 (present) and 0 (absent). Because SONUS records negative data as blank values, it was difficult to ascertain whether values were truly negative or missing. Any clinic with a series of blanks for any particular neurologic exam variable was considered to have missing data, and the entire clinic was excluded during this process (n = 1,160, five clinics). The data set used in the analysis consisted of samples from seven clinics in Florida, Pennsylvania, Texas, and Utah.

In summary, inclusion criteria were (a) clinics that have recorded neurologic exam findings in the database; (b) adult patients (≥18 years old); (c) having at least one neurologic exam finding (+/-); and (d) having radiographic finding (+/-). The exclusion criteria were (a) clinics that did not record their exam findings in the database; (b) children (<18 years old); (c) age could not be calculated due to missing date of birth or exam date; and (d) age error (≤0).

**Figure 1:**
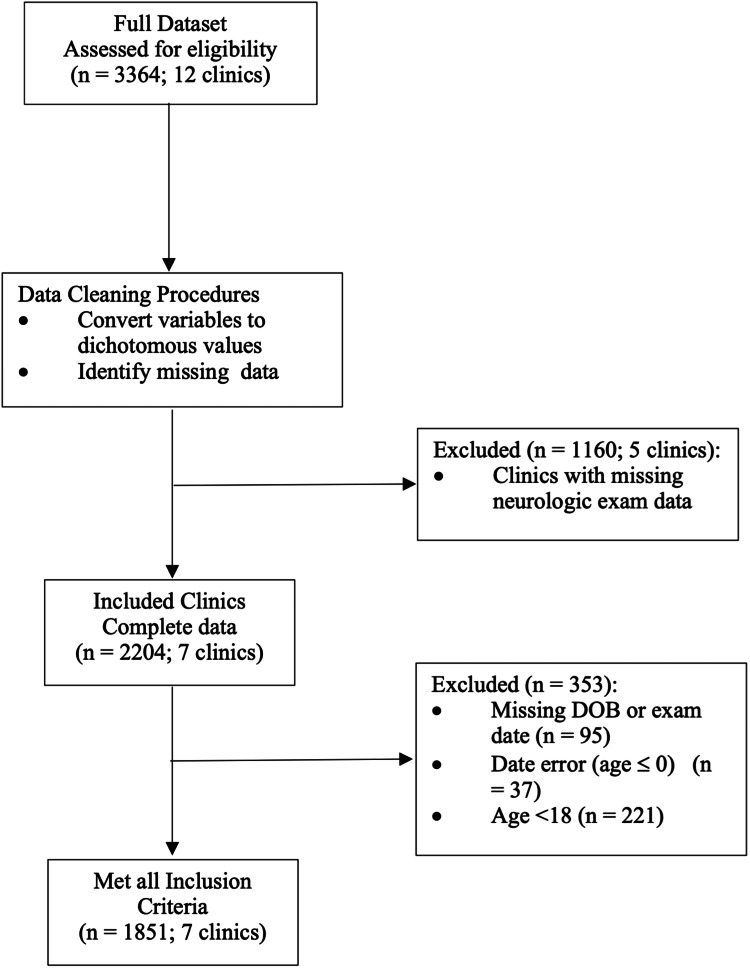
Flow diagram.

After applying the inclusion and exclusion criteria, 1,851 subjects from seven clinics were used in this analysis (Figure 1); 1,513 (45%) patients were removed. Gender was available for 1,435 patients from six clinics.

Variables of interest

Initial examination procedures included analysis based on physical and radiographic findings. Physical exam included testing of the following five neurological indicators: (1) palpation for inflammation of the C2 DRG (DRG); (2) supine leg length inequality (SLC), (3) Fakuda Step test (Fakuda), (4) right or left arm weakness (WA), and (5) lumbar muscle tautness (LB). All physical exam findings were analyzed as dichotomous variables, 1 (present)/0 (absent). After the data cleaning procedures described above (Figure 1), all remaining data were considered complete, and no blanks were further treated as missing data.

**Figure 2:**
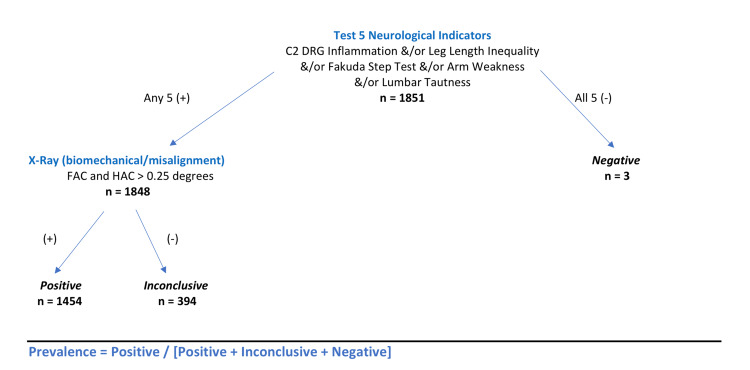
Vertebral subluxation prevalence algorithm. DRG = dorsal root ganglion; FAC = frontal atlas cranium line; HAC = horizontal atlas cranium line

Radiographic analysis was used to determine the biomechanical misalignment of the upper cervical spine, based on a combination of two measurements, FAC and HAC (FHAC). A cutoff measurement value of 0.25 degrees was used for both X-ray mensuration to account for measurement error, anatomical variants, and any other potential anomalies, as per clinic protocol. FHAC was treated as a dichotomous variable as well; any measurements above the cutoff were analyzed as 1 (present) and values below the cutoff were given a 0 (absent) value. Figure 2 shows the algorithm used to determine prevalence; a formula is also included for easy reference. The algorithm reflects the operational definition of VS used in this study.

Patients were classified as positive (for subluxation) if they met two criteria. Criteria 1 was a presentation with a minimum of one neurological indicator of VS: (a) C2 DRG tenderness; OR (b) positive Fakuda Step test; OR (c) leg length inequality; OR (d) tautness of the erector spinae muscles; OR (e) upper extremity muscle weakness. Criteria 2 was a presentation with the biomechanical indicator in conjunction with a neurological finding, based on (a) FAC >0.25 degrees, AND (b) HAC >0.25 degrees.

Algorithm criteria (Figure 2) required that one neurological indicator (DRG, Fakuda, SLC, LB, or WA) AND misalignment on X-ray must be present to establish the presence of subluxation.

Patients who did not meet the first criteria were considered negative, as they did not have any neurological indicators; no further testing was indicated, and no X-ray data were compiled for these individuals. Patients who were positive for the first criteria but did not meet the second criteria on X-ray were classified as inconclusive. Although they demonstrated signs of potential neurological dysfunction, no kinesiologic dysfunction was seen at the spinal level of interest for this protocol.

In addition to the overall analysis of the sample, age-stratified analysis was performed for the following four groups: (1) 18-30 years (n = 534); (2) 31-50 years (n = 675); (3) 51-70 years (n = 508); (4) >70 years (n = 134). A gender-stratified analysis was also performed (n = 1,435).

Analysis

SAS Enterprise Guide (SAS Institute Inc., Cary NC) and Microsoft Excel were both used to run the analyses of descriptive statistics and associated confidence intervals. Patient demographic data were summarized for age, gender, and past health history characteristics of the overall sample as well as per clinic. The prevalence of VS following the algorithm presented in Figure 2 was estimated. Sex-stratified and age-stratified analyses for each of the clinics were also performed.

Ethics statement

Based on the nature of this study, the Emory University Institutional Review Board determined that no further IRB review was required.

## Results

Characteristics of the sample

A total of 1,851 patient records were utilized in this analysis (Figure 1). Table 1 provides a summary of the patient sample demographics. The mean age of patients was 43.48 (SD = 16.8; range = 18-91 years). More females (n = 927, 64.6%) than males (n = 508, 35.4%) presented for chiropractic care.

**Table 1:** Patient demographics and reason for chiropractic care. *: n = 1,435. ^a^: Includes neck, mid-, low-back, or sacroiliac pain, possibly due to disc involvement. ^b^: Includes scoliosis and antero- or retro-listhesis. ^c^: Includes paraesthesia, sciatica, cramping, spasm, or foot drop. ^d^: Includes head pain/pressure or headaches of all types, including migraines. ^e^: Includes jaw complaints or any extremity or joint pain. ^f^: Includes all ear, nose, or throat-related complaints including cranial nerve involvement. ^g^: Includes neurological (i.e., seizures, tremors, Parkinson’s disease), cognitive (i.e., dementia, memory loss), or concussion/whiplash/post-concussive issues. ^h^: Includes anxiety, stress, depression, mood swings, or brain fog. ^i^: Includes cardiovascular, endocrine, respiratory, gastrointestinal, genitourinary, or reproductive conditions. ^j^: Includes issues with fatigue, sleep difficulty, weight change, or energy levels. ^k^: Includes other past diagnoses such as pregnancy, Lyme disease, past history of stroke, or fibromyalgia.

	Clinic A		Clinic B		Clinic C		Clinic D		Clinic E		Clinic F		Clinic G		Total	
	Count	%	Count	%	Count	%	Count	%	Count	%	Count	%	Count	%	Count	%
Total sample size	59		117		125		92		883		129		446		1,851	
Age 18–30 (n)	14	23.7%	14	12.0%	49	39.2%	21	22.8%	347	39.3%	24	18.6%	65	14.6%	534	28.8%
Age 31–50 (n)	18	30.5%	61	52.1%	48	38.4%	44	47.8%	309	35.0%	46	35.7%	149	33.4%	675	36.5%
Age 51–70 (n)	22	37.3%	36	30.8%	27	21.6%	19	20.7%	178	20.2%	52	40.3%	174	39.0%	508	27.4%
Age >70 (n)	5	8.5%	6	5.1%	1	0.8%	8	8.7%	49	5.5%	7	5.4%	58	13.0%	134	7.2%
Mean age (SD)	49.37 (17.2)		47.28 (14.7)		37.9 (13.9)		43.45 (16.2)		39.57 (16.3)		46.55 (15.5)		50.11 (16.8)		43.48 (16.8)	
% Female*	36	61.0%	35	29.9%	81	64.8%	--	--	478	54.1%	37	28.7%	260	58.3%	927	64.6%
MVA	0		1		10		1		0		0		0		12	0.65%
Chief complaints																
Pain^a^	0		65		13		66		2		92		3		241	13.0%
Postural^b^	0		1		0		0		0		7		3		11	0.6%
Neuropathy^c^	2		11		17		1		84		10		3		128	6.9%
HA^d^	0		25		4		30		1		15		3		78	4.2%
Extremity^e^	0		20		9		9		7		9		3		57	3.1%
ENT^f^	5		8		30		4		75		8		3		133	7.2%
NeuroCog^g^	0		1		3		3		19		1		3		30	1.6%
Mental^h^	0		2		44		1		95		1		3		146	7.9%
Visceral^i^	0		4		16		0		48		2		3		73	3.9%
General^j^	0		3		27		0		47		3		3		83	4.5%
Other^k^	1		3		5		2		14		2		3		30	1.6%
Wellness	0		2		0		0		0		0		3		5	0.3%
None	53		30		46		29		593		33		425		1209	65.3%

A broad number of presenting symptoms were recorded as the reason for chiropractic care in subjects (n = 642), and many patients reported more than one complaint. Patient-reported reasons for presenting to the chiropractor included various forms of spinal or extremity pain, numbness, or tingling; headaches; ear, nose, and throat-related symptoms or conditions; visceral symptoms; mental health concerns; neurocognitive issues; as well as concerns about general health. Two subjects cited overall wellness, and no chief complaint was recorded for 1,209 (65.3%) subjects. In total, 12 (0.65%) subjects reported a history of a motor vehicle accident.

Analysis by clinic was also performed. The mean age was lowest in clinic C (37.9 years) and highest in clinic G (50.11 years). Clinic C had the largest number of females (64.8%), and clinic F had the smallest (28.7%). No gender information was available for subjects from clinic D.

Key findings

Table 2 provides the percent prevalence of positive subjects, along with its 95% confidence interval. Large variations in prevalence are seen between clinics (50-93.88%), and the sample sizes also differ (range = 59-883 patients). The prevalence of VS among males (80.15%, n = 508) is similar to that of females (77.17%, n = 927). Prevalence by age demonstrated an inverse relationship with age; the 18-30 group (n = 534) demonstrated 86.33% prevalence, while the group of patients over 70 years of age (n = 134) had a 73.88% prevalence.

**Table 2:** Prevalence of vertebral subluxation.

	N	Prevalence
Overall (95% CI)	1851	78.55% (76.68, 80.42)
Clinic A	59	86.44%
Clinic B	117	89.74%
Clinic C	125	93.60%
Clinic D	92	50.00%
Clinic E	883	93.88%
Clinic F	129	62.79%
Clinic G	446	50.45%
Gender		
Female	927	77.17%
Male	508	80.15%
Age group		
18–30	534	86.33%
31–50	675	76.44%
51–70	508	74.41%
>70	134	73.88%

When age-stratified prevalence was performed in each clinic (Table 3), no clear relationship between age group and prevalence was observed. The prevalence of VS for each clinic and age-stratified group was ≥50% except in two 31-50-year-old subgroups from clinics D and G (40.91% and 44.30% prevalence, respectively).

**Table 3:** Prevalence of vertebral subluxation, per clinic and age-stratified. Age-stratified prevalence = positive/total (% prevalence).

Clinic (n = sample size)	Age 18–30	Age 31–50	Age 51–70	Age >70	Overall prevalence
A (n = 59)	12/14 (85.71%)	17/18 (94.44%)	17/22 (77.27%)	5/5 (100.00%)	51/59 (86.44%)
B (n = 117)	12/15 (85.71%)	56/61 (91.80%)	31/36 (86.11%)	6/6 (100.00%)	105/117 (89.74%)
C (n = 125)	48/49 (97.96%)	45/48 (93.75%)	23/27 (85.19%)	1/1 (100.00%)	117/125 (93.60%)
D (n = 92)	12/21 (57.14%)	18/44 (40.91%)	11/19 (57.89%)	5/8 (62.50%)	46/92 (50.00%)
E (n = 883)	324/347 (93.37%)	288/309 (93.20%)	170/178 (95.51%)	47/49 (95.92%)	829/883 (93.88%)
F (n = 129)	14/24 (58.33%)	26/46 (56.52%)	35/52 (67.31%)	6/7 (85.71%)	81/129 (62.79%)
G (n = 446)	39/65 (60.00%)	66/149 (44.30%)	91/174 (52.30%)	29/58 (50.00%)	225/446 (50.45%)
All clinics (n = 1,851)	461/534 (86.33%)	516/675 (76.44%)	378/508 (74.41%)	99/134 (73.88%)	1,454/1,851 (78.55%)

Summary findings

The overall prevalence of radiographic and neurological findings indicating the presence of VS was found to be 78.55% (95% CI = 76.68%-80.42%). Prevalence of VS was higher in younger groups (ages 18-30 = 86.33%) and was decreasing with age (>70 years = 73.88%) in the overall sample. There was greater age-stratified variation at the per-clinic level; however, a high prevalence of VS was seen in all older age groups. Per clinic, >95% prevalence was seen in four of seven clinics for the >70 group, one of seven clinics for ages 51-70, zero of seven clinics for ages 31-50, and one of seven clinics for ages 18-30 (Table 3). When the data are viewed this way, a possible increasing prevalence in older age groups emerges.

**Figure 3:**
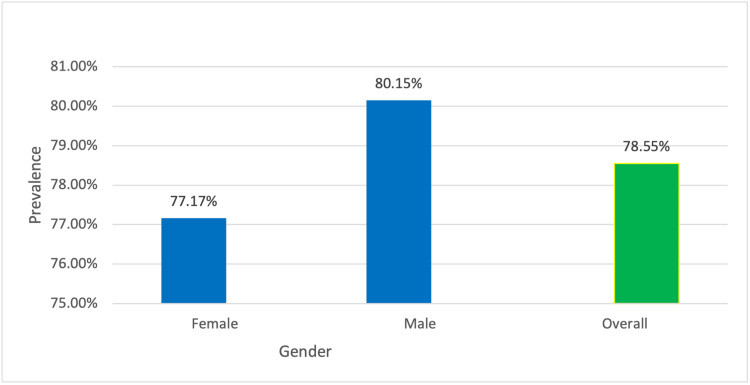
Prevalence of vertebral subluxation by gender.

Prevalence of VS for females (77.17%, 95% CI = 78.33%-81.97%) was similar to that of males (80.15%, 95% CI = 75.25%-79.08%) (Figure 3).

## Discussion

Study summary

A multi-clinic dataset of 3,364 patients presenting for chiropractic care was analyzed to estimate the prevalence of VS. After data cleaning procedures, data from 1,851 subjects were available for overall, age-stratified, and gender-stratified analysis. The prevalence of VS was calculated using an algorithm that requires two criteria, i.e., the presence of at least one neurologic indicator (DRG, SLC, Fakuda, WA, or LB) and a minimum cutoff value for the radiographic findings (FHAC).

The overall prevalence of VS using data from seven clinics was 78.55% (95% CI = 76.68%-80.42%). Notably, ≥50% prevalence was found for each individual clinic, although variations were observed between clinics. Prevalence by gender was 77.17% for females (715 of 927) and 80.15% for males (407 of 508). The data in this study are consistent with previously published work in that women are more likely to visit a chiropractor [8]. However, the high prevalence of VS found in this study suggests that it may be of equal importance that men also seek chiropractic evaluation. There are both environmental and biological factors that influence gender prevalence in health [24]. However, in the sequelae of disease processes, each of these factors possibly aggravates the progression of a disease. VS is not a disease entity but rather a condition of reduced functional efficiency of the nervous system [6]; the presence of VS could lay the foreground for declining health.

A broad number of chief complaints were cited by patients as their reasons for seeking chiropractic care. These included various forms of spinal or extremity pain, numbness, or tingling; headaches; ear, nose, and throat-related symptoms or conditions; visceral symptoms; mental health concerns; neurocognitive issues; as well as concerns about general health. Two subjects cited overall wellness, and no chief complaint was recorded for 1,209 (65.3%) subjects. Twelve (0.65%) subjects reported a history of a motor vehicle accident. The data suggests that chiropractic patients may or may not be symptomatic at initial presentation, and some may experience subclinical benefits from chiropractic care. Follow-up analysis on the resolution of patient-reported symptomatology along with a reduction in subluxation indicators from chiropractic care may help elucidate the impact of VS on health promotion and prevention.

Furthermore, prevalence by age was found to decrease with increasing age in the overall sample. However, this seemingly inverse relationship between age and prevalence of subluxation did not persist within individual clinics and may thus constitute an incidental finding. Notably, in six of the seven clinics, the highest prevalence was still seen in the oldest age category (n = 134). Overall, 95% or higher prevalence was seen at the highest frequency in this group, i.e., in four of seven clinics for >70 years of age, one of seven clinics for ages 51-70, zero of seven clinics for ages 31-50, and one of seven clinics for ages 18-30. It is commonly known that there is an exponential rise in mortality rates with aging [25]. It is possible that older populations with subclinical symptomatology are presenting to a chiropractor. The literature shows that health-promoting activities reduce morbidity at older ages even without an impact on population life expectancy [25]. Chiropractic management of VS at younger ages may prevent further health challenges at later stages in life. As seen with this analysis, older individuals exhibit a greater prevalence of unmanaged VS, and as seen in the published literature, older populations generally experience greater morbidity. The implication of these findings is that quantifying the prevalence of subluxation may play a key role in longevity and quality of life through health promotion and disease prevention. The WHO defines health as “a state of complete physical, mental and social well-being and not merely the absence of disease or infirmity” [26]. Chiropractic care is associated with improvement in multiple health domains, including physical state, mental/emotional state, stress evaluation, and life enjoyment [9]. Furthermore, quality of life is improved through chiropractic care alongside health promotion [9].

A subset of the sample (n = 394) was classified as inconclusive, alongside positive (n = 1,454) and negative (n = 3) individuals. This subgroup is significant in its role as the numerator and denominator used to calculate prevalence. The inconclusive group is defined as subjects who have at least one neurological indicator but do not meet the criteria set forth for the biomechanical component of the operational definition for VS (Figure 2). However, the radiographic analysis was limited to two criteria, FAC and HAC, with a cutoff value of >0.25 degrees to account for genetic anomalies in the individual cervical spines. Incorporating additional radiographic criteria or lines of mensuration in the operational definition could significantly increase the prevalence calculations presented in this study.

As outlined in Figure 1, data from five clinics (n = 1,160) were excluded from this analysis due to missing neurological exam data. This relatively large subset of the data potentially affects prevalence estimates. Some of these groups of patients had radiographic exam data recorded in SONUS. Thus, it may be presumed that they met neurological criteria according to the VS algorithm presented in Figure 2. However, that exam information was not uploaded to SONUS by specific clinics. Inclusion on the basis of the radiographic criteria alone may have increased prevalence estimates reported on the entire dataset.

Overall, it is important to note a high prevalence of VS in all subgroups as well as the total sample based on the operational definition adopted in this study. Although there was variability of findings between clinics, all clinics showed agreement that over half of the subjects examined were positive for VS (50.45-93.88%). Data from four of seven clinics revealed >95% prevalence in the oldest age group. All but two age and clinic-stratified subgroups reported ≥50%, further suggesting the high prevalence of VS in the general population.

Strengths and limitations

This is the first study to date that has attempted to estimate the prevalence of VS using a large sample from a multi-clinic dataset. Prevalence estimates are highly dependent on the adopted operational definition, which must be concrete, evidence-based, and comprehensive. An operational definition should include valid measurements and processes that reduce the likelihood of false positives and false negatives. In this case, an operational definition that incorporated a number of direct measures of neurologic and kinesiologic subluxation components commonly used in chiropractic clinical practice was used as the basis for analysis. Previous studies reported anecdotal prevalence estimates based only on neurological findings or biomechanical measures in small samples.

Although the size and broad geographic distribution across seven clinics, six cities, and four states are strengths of the study, the dataset was derived from a population that has already presented for chiropractic care. This suggests possible selection bias; that is, subjects may not be representative of the general population. They may have been symptomatic or had some other driving reason to be analyzed for VS in the first place.

Missing data was an additional limitation in this dataset. When fields were left blank, this omission was deemed intentional and meant to indicate a null value. However, if a particular field was blank for the majority of patients from a specific clinic, it was assumed to be missing data rather than negative findings; these clinics were excluded from the analysis. As discussed previously, it is likely that missing data affected the prevalence estimates. Data on occupational status and physical activity were unavailable for this analysis, although both factors could play a role in spinal health. Errors in age (≤0 years) were also excluded from the analysis. Furthermore, only data on cervical vertebrae radiographs were utilized in this study; VS indicators can also be found at thoracic and lumbar levels. Such data could suggest a higher prevalence in the general population.

The calculation for the confidence interval of the overall sample prevalence assumed each patient as independent. However, the clinic-stratified analysis revealed a clinic effect - findings for each clinic and its clustered sample are different. A clustering effect is possible and may warrant consideration in confidence interval calculations for the overall prevalence estimate.

It would be presumed that little geographic variation of subluxation prevalence would occur, but there were significant variations per clinic in this dataset. This could have been due to data entry issues but was likely influenced by variance in the sample size from each clinic. Data from clinics with larger sample sizes can dominate the analysis and create statistical artifacts at the macroscopic level that vary from what is seen when examining the same data by individual clinics. Although there was a high level of variability in prevalence between clinics, all clinics showed agreement that over half of the subjects examined were positive for VS (50.45-93.88%). Gender data were unavailable from one clinic.

Finally, 1,208 of the subjects have no chief complaint of record. Since “wellness” is a possible option for visiting the chiropractor, we could not assume that a blank field corresponded to an asymptomatic patient. Therefore, representative estimates of prevalence based on chief complaints could not be calculated from Table 1.

## Conclusions

The results of this study suggest a high rate of prevalence of VS in the study population. Despite the inherent limitations of this limited sample, the broad geographic distribution of the clinics suggests these findings hold some generalizability and may be representative of the general population. This is the first study of its magnitude and application of an operational definition to estimate the prevalence of VS. Further investigation into the epidemiology of vertebral subluxation is warranted to strengthen inferences about the role of subluxation in health and longevity. More robust data collection in multiple clinics using the same assessment tools is recommended to expand this knowledge base. There may be a need to evaluate and expand the operational definition of VS based on reliable and valid criteria. Consideration should be given to whether additional radiographic analysis is warranted in the inconclusive group that presents with neurological findings but does not meet the criteria for misalignment by the operational definition adopted in this study. Future studies should examine longitudinal outcomes of care to further elucidate the public health implications of the prevalence of VS.
